# Pesticide detection combining the Wasserstein generative adversarial network and the residual neural network based on terahertz spectroscopy

**DOI:** 10.1039/d1ra06905e

**Published:** 2022-01-11

**Authors:** Ruizhao Yang, Yun Li, Binyi Qin, Di Zhao, Yongjin Gan, Jincun Zheng

**Affiliations:** School of Physics and Telecommunication Engineering, Yulin Normal University Yulin China qby207@163.com; Guangxi Colleges and Universities Key Laboratory of Complex System Optimization and Big Data Processing, Yulin Normal University Yulin China; College of Chemistry and Food Science, Yulin Normal University Yulin China

## Abstract

Feature extraction is a key factor to detect pesticides using terahertz spectroscopy. Compared to traditional methods, deep learning is able to obtain better insights into complex data features at high levels of abstraction. However, reports about the application of deep learning in THz spectroscopy are rare. The main limitation of deep learning to analyse terahertz spectroscopy is insufficient learning samples. In this study, we proposed a WGAN-ResNet method, which combines two deep learning networks, the Wasserstein generative adversarial network (WGAN) and the residual neural network (ResNet), to detect carbendazim based on terahertz spectroscopy. The Wasserstein generative adversarial network and pretraining model technology were employed to solve the problem of insufficient learning samples for training the ResNet. The Wasserstein generative adversarial network was used for generating more new learning samples. At the same time, pretraining model technology was applied to reduce the training parameters, in order to avoid residual neural network overfitting. The results demonstrate that our proposed method achieves a 91.4% accuracy rate, which is better than those of support vector machine, *k*-nearest neighbor, naïve Bayes model and ensemble learning. In summary, our proposed method demonstrates the potential application of deep learning in pesticide residue detection, expanding the application of THz spectroscopy.

## Abstract 2

We proposed a WGAN-ResNet method, which combines two deep learning networks, the Wasserstein generative adversarial network (WGAN) and residual neural network (ResNet), to detect carbendazim based on terahertz spectroscopy.

## Graphical abstract

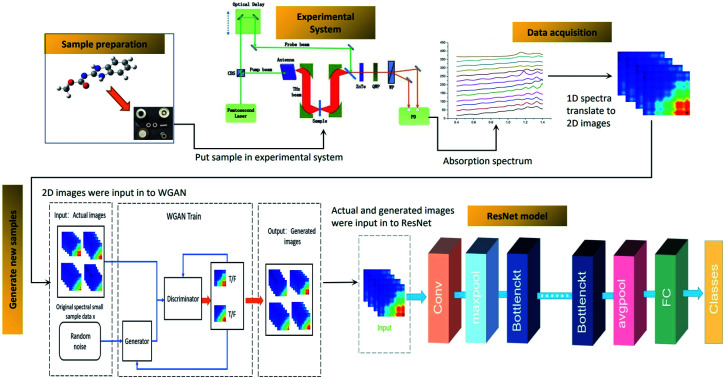

## 1 Introduction

The problem of pesticide residues has always been a top priority for the public. The excessive use of pesticides in crop planting leads to the frequent occurrence of pesticide residues in crop products, which threatens human health. Accurate and rapid pesticide residue detection is of great significance for food quality and safety control. High-performance liquid chromatography,^1,2^ gas chromatography,^3^ capillary electrophoresis^4^ and immunoassay techniques^5^ are common methods for the detection of pesticide residues. However, these methods are destructive, harmful solvents are required and they are time-consuming.^6–8^ Therefore, a rapid and effective analytical method is urgently needed for the determination of pesticide residues.

Terahertz (THz) spectroscopy is considered to be a promising detection method due to its low-energy, high resolution and penetrability.^9–11^ Because it is sensitive to the vibrational modes, THz spectra contain abundant useful information on the vibrational modes of the target. In recent years, some researchers have combined THz fingerprints and chemometric techniques to detect foreign bodies,^12,13^ toxic and harmful compounds,^10,14,15^ pesticides,^16–18^ antibiotics,^19,20^ microorganisms^21–23^ and adulteration.^24^ The above studies show that data feature extraction is a key factor affecting the detection results. Compared to traditional methods, deep learning is able to obtain better insights into complex data features at high levels of abstraction. The residual neural network (ResNet), proposed by He,^25^ is a deep learning network, which solves the degradation issues of traditional deep convolutional networks. It has been applied in the real-time quality assessment of pediatric MRI images,^26^ the clinical diagnosis of COVID-19 patients,^27^ the identification of cashmere and sheep wool fibers,^28^ rotating machinery fault diagnosis^29^ and so on. However, there are few reports about the application of deep learning in THz spectroscopy. The main reason is that learning samples of THz spectra are too few to meet deep learning requirements. It is known that the learning results are worse when a deep learning model is short of learning samples. Measuring more THz spectra data is one way to solve the problem of insufficient learning samples. But it is not a good approach, because it demands a higher cost and more time.

The generative adversarial network (GAN) is a sample generation model, which was proposed by Goodfellow^30^ in 2014. It is able to generate new samples with the same distribution as real samples to expand the size of labeled samples.^31^ The GAN contains a generator and a discriminator. The GAN learns the distribution of real samples during the game between a generator and a discriminator. In the process of GAN training, the generator tries its best to fit the distribution of real samples and generate new samples, while the discriminator tries its best to distinguish real samples from new samples. In recent years, GAN has been used for generating new conversation data,^32^ new samples for the minority class of various imbalanced datasets^33^ and new high-quality images.^34^ As there is a shortage of training data, it is difficult to train a learning model from scratch. Fine-tuning a deep learning model, which has been pretrained using a large set of labeled natural images, is a promising method to solve the shortage of learning samples. It has been applied successfully to various computer vision tasks such as food image recognition,^35^ mammogram image classification^36^ and multi-label legal document classification.^37^

Carbendazim is a type of broad-spectrum benzimidazole fungicide, which has been commonly employed to control plant diseases in cereals and fruits. Rice is an important food crop for human beings, which a wide area has been cultivated for. A large amount of carbendazim is used in the prevention of rice blast and rice sheath blight fungus. Studies have shown that high doses of carbendazim can damage testicles, which causes infertility.^38,39^ In this study, we proposed the WGAN-ResNet method, which combines two deep learning networks, the Wasserstein generative adversarial network (WGAN) and the residual neural network (ResNet), to detect carbendazim based on THz spectroscopy. The WGAN was employed to generate new learning samples, which solves the problem of learning results being worse caused by insufficient learning samples. The ResNet was applied to quantify different concentrations of carbendazim samples. At the same time, pretraining model technology was employed to reduce the training parameters in the ResNet. The results demonstrate that our proposed method shows the potential application of deep learning in pesticide residue detection, expanding the application of THz spectroscopy.

## 2 Experimental and theoretical methods

### 2.1 Experimental system and procedure

**Fig. 1:**
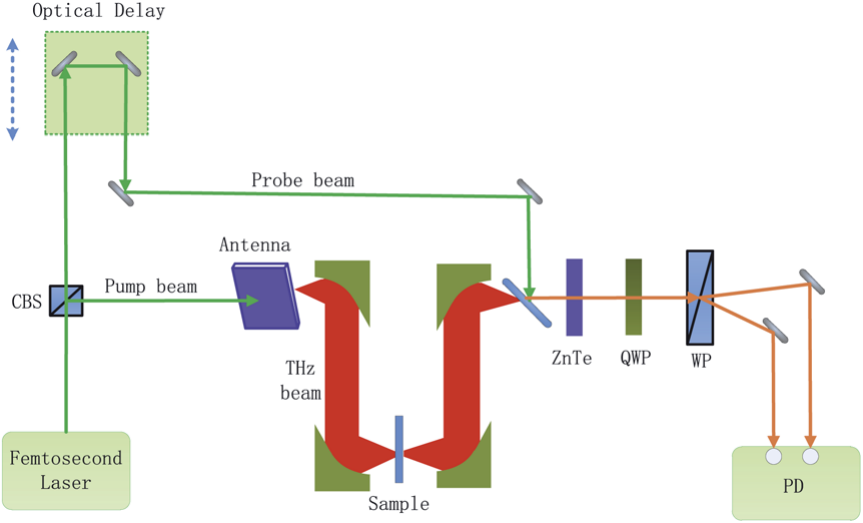
Schematic of the experimental system.

The composition of the experimental system is shown in Fig. 1. A femtosecond laser beam (with a pulse width of about 100 fs and a wavelength centered around 780 nm) is generated by an ultra-fast fiber laser. As the laser beam goes through a cubic beam splitter, it is divided into a pump beam and a probe beam. The THz beam is elicited as the pump beam, which is concentrated on a photoconductive antenna. After the THz beam goes across the sample, it carries the sample information. When the THz beam encounters the probe beam at the ZnTe crystal, the probe beam is modulated by the THz beam. And then, the modulated probe beam goes through a quarter-wave-plate (QWP) and a Wollaston prism (WP). Finally, the modulated probe beam is then detected by a set of balanced photodiodes. To reduce the absorption of the THz signal by atmospheric water vapor, the THz beam path is enclosed in a dry air purged box.

**Fig. 2:**
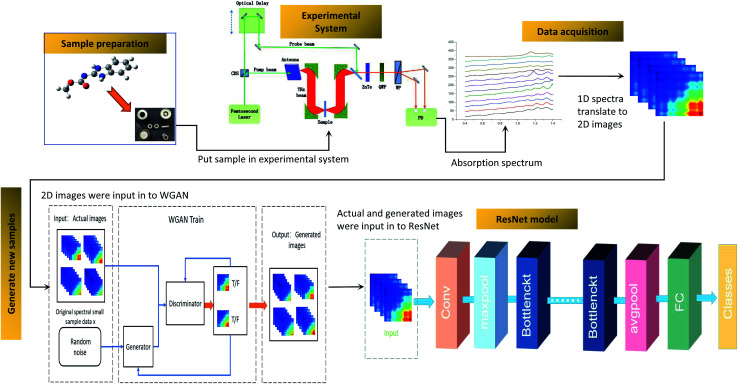
Flow chart of the detection of carbendazim using WGAN-ResNet based on THz spectroscopy.

Fig. 2 shows the flow chart for the whole process. It includes the preparation of the sample, data acquisition, generating new samples and the ResNet model. Firstly, samples were made into tablets after drying and sieving. Secondly, the absorption coefficients of the samples were calculated by using the THz time-domain spectra, and then the absorption coefficients were translated to two-dimensional images. Thirdly, new samples were generated by WGAN to increase the training samples. Finally, ResNet was trained and employed to quantify the samples. The details of these procedures are described in the following sections.

### 2.2 Sample preparation

Carbendazim powder, with a purity of 98%, was purchased from Adamas and used without further purification. Rice powder was purchased from a local market. First, carbendazim and rice powder were put into a vacuum drying oven. The temperature was set to 323 K, and the drying time was set to 1 hour. Then, the two powders were separately sieved with a 100 mesh sieve. After that, sample pellets, mixed with different concentrations of carbendazim and rice, were pressed into 1 mm thick tablets using a hydraulic press. Finally, 13 concentrations of carbendazim samples (0%, 2%, 4%, 6%, 8%, 10%, 15%, 20%, 25%, 30%, 40%, 50% and 100%) were prepared. The total number of samples was 429, including 33 samples of each concentration.

### 2.3 Data acquisition

In the experiment, the THz time-domain spectrum of dry air was used as a reference signal. To address random noise, we carried out the measurements of each reference and each sample three times. When the time-domain spectra underwent fast Fourier transformation, the amplitude and phase in the frequency domain were obtained. The samples’ refractive indices *n*(*ω*) and absorption coefficients *α*(*ω*) were calculated based on the amplitude and phase.^40,41^1
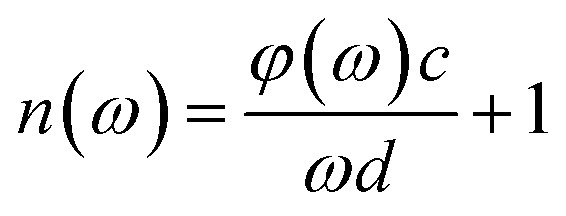
2
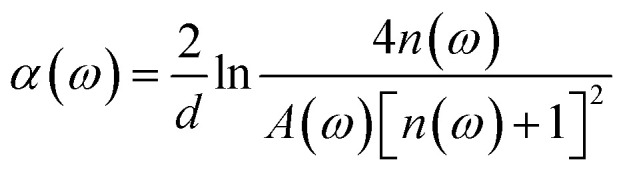
where *ω* is the frequency, *c* is the speed of light, *d* is the thickness of the sample, *φ*(*ω*) is the phase difference of the sample and reference, and *A*(*ω*) is the amplitude ratio of the sample and reference.

The depth of the network is very important for the performance of the model. When the number of network layers is increased, the network can extract more complex feature patterns. But deep networks present a degradation problem: when the network depth increases, the network accuracy becomes saturated or even decreases. The ResNet is able to solve the degradation problem as the network depth increases. The ResNet is good at two-dimensional image recognition tasks.^29,42–44^ To use our proposed WGAN-ResNet analysis with the THz spectrum, we firstly translated a one-dimensional absorption coefficient to a two-dimensional image as follows,3
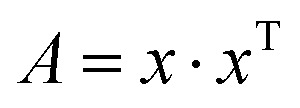
where *x* is the sample absorption coefficient, which is a *n*-dimensional column vector, and *x*^T^ is a transpose of *x*. Thus, *A* is a *n* × *n* size image.

After the above calculation, we obtained 429 images. These images were called actual images. Then, these actual images were put into the WGAN to generate 13 concentrations gradients of new image samples (0%, 2%, 4%, 6%, 8%, 10%, 15%, 20%, 25%, 30%, 40%, 50% and 100%). Each concentration was 3495 new image samples. To distinguish between new images and actual images, we called the new images generated images.

### 2.4 Generating new samples

To address the shortage of learning samples, we employed the WGAN for data augmentation. WGAN was proposed by Martin Arjovsky. He introduced the Earth-Mover (EM) distance, instead of KL divergence or JS divergence,^45^ to address the issue of GANs being hard to train.^46^ The EM distance is also called Wasserstein-1, which is defined by:4
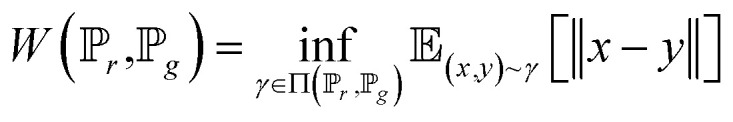
where 
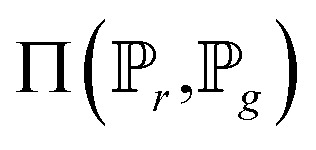
 is the set of all joint distributions *γ*(*x*,*y*), of which the marginals are 
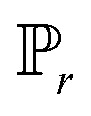
 and 
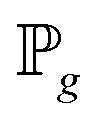
, respectively.

Eqn (4) can be translated based on the Kantorovich–Rubinstein duality:5
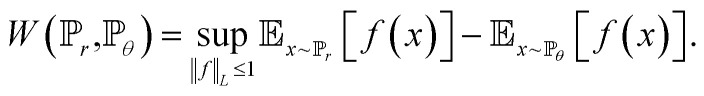
As ‖*f*‖_*L*_ ≤ 1 is replaced by ‖*f*‖_*L*_ ≤ *K*, eqn (5) can be rewritten as:6
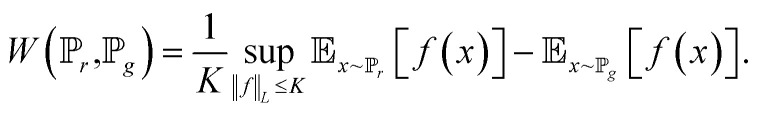
If we have a parameterized family of functions, 
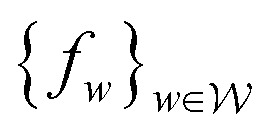
, that are all *K*-Lipschitz for some *K*, we could consider solving the problem:7
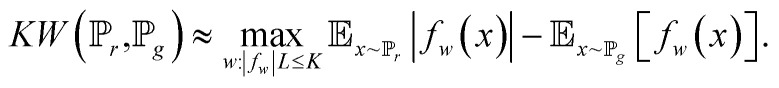
Thus, the generator loss function of WGAN is:8
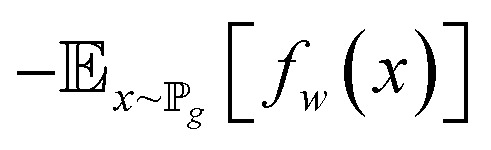
and the discriminator loss function of WGAN is:9
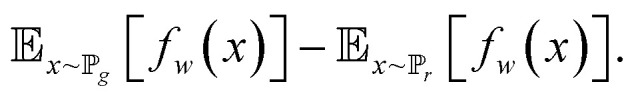


**Fig. 3:**
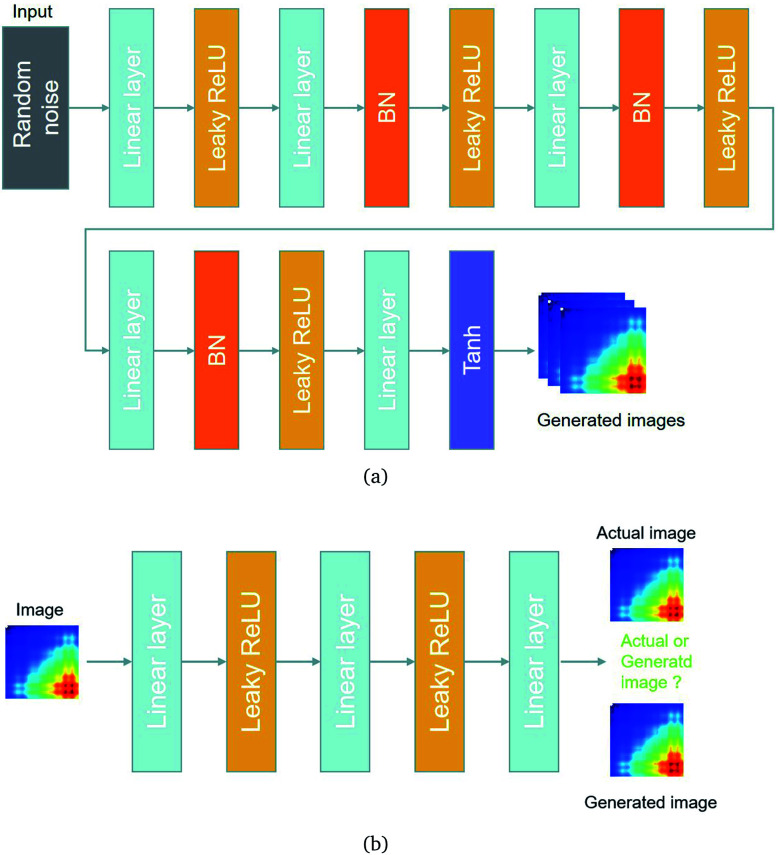
The architectures of the generator and a discriminator in the WGAN. (a) The architecture of the generator. (b) The architecture of the discriminator.

The architectures of the generator and discriminator in the WGAN are illustrated in Fig. 3. The generated images were produced by the generator, when random noise was input into the generator. And then an image was decided to be either an actual image or a generated image by the discriminator. The generator was trained to generate images which are more similar to actual images, and the discriminator was also trained to discriminate between images more accurately. The generator and the discriminator were adversarial with each other. When the discriminator could not make a decision on whether an image is a generated image or an actual image, the training was finished.

### 2.5 ResNet model

A deep network can obtain better feature extraction capability than a shallow network. However, a degradation problem will occur when the network depth increases. To deal with the degradation problem, He *et al.* proposed the ResNet.^25^ They make these stacked layers fit a residual mapping, taking the place of making these layers fit a desired underlying mapping.

**Fig. 4:**
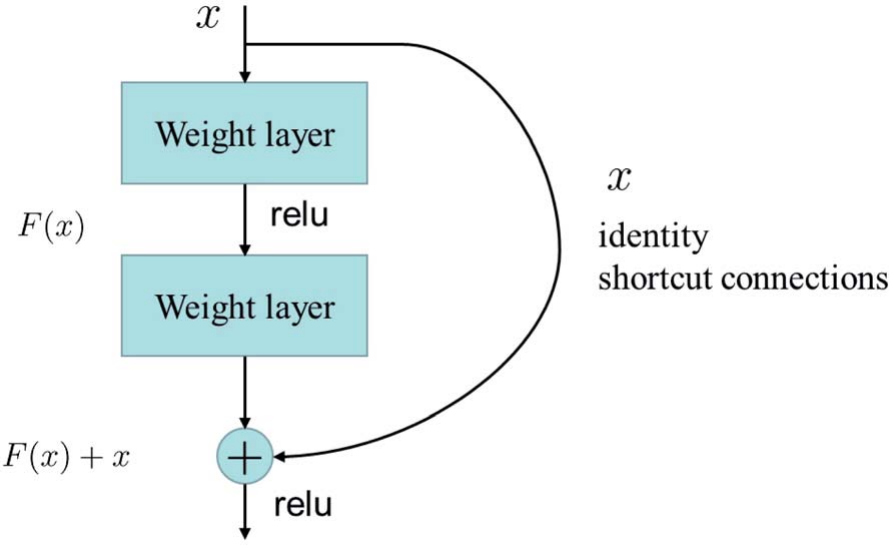
Residual learning: a building block from ref. 25.

Let the desired underlying mapping be denoted as *H*(*x*), where *x* denotes the input of the first of these layers. The stacked nonlinear layers fit another mapping of *F*(*x*)≔*H*(*x*) − *x*. So, the original mapping is recast into *F*(*x*) + *x*. At last, the formulation of *F*(*x*) + *x* can be realized by feedforward neural networks with identity shortcut connections, shown in Fig. 4.

In this study, we selected two ResNets (an 18 layer ResNet and a 152 layer ResNet). To satisfy our classification task, we changed the last fully connected layer output of the original ResNet from 1000 to 13. The network architecture of the 18 layer ResNet and 152 layer ResNet are listed in Table 1. The number beside the bracket represents the number of blocks stacked. Down-sampling was performed by conv3_1, conv4_1, and conv5_1 with a stride of 2.

**Table 1:** Architectures of the two ResNets

Layer name	Output size	18 layer ResNet	152 layer ResNet
conv1	112 × 112	7 × 7.64, stride 2	
conv2_x	56 × 56	3 × 3 max pool, stride 2	
		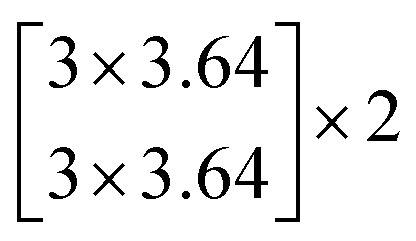	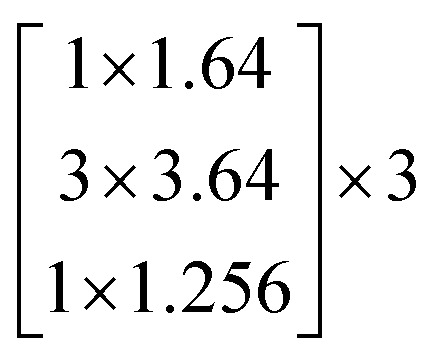
conv3_x	28 × 28	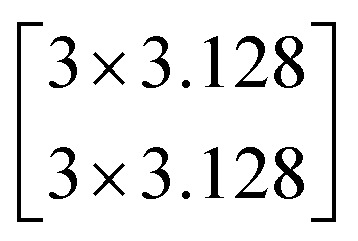	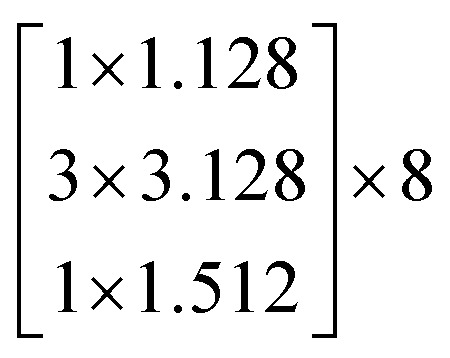
conv4_x	14 × 14	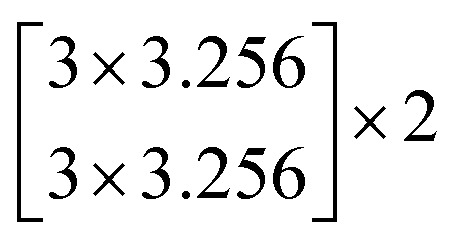	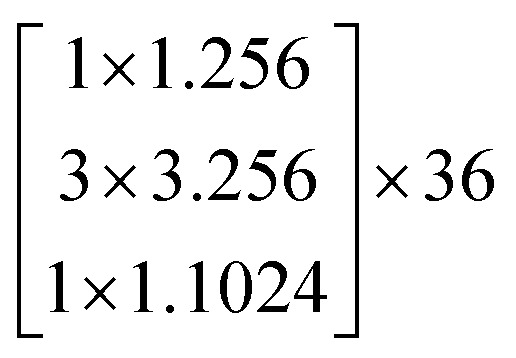
conv5_x	7 × 7	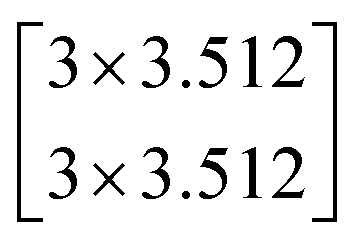	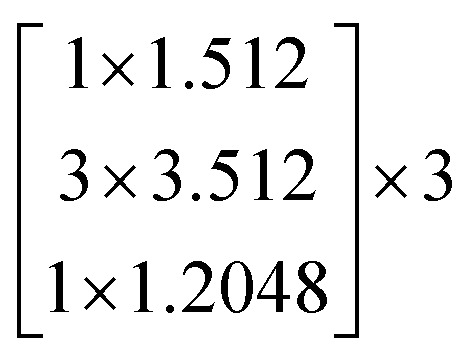
fc	1 × 1	Average pool, 13-d fc, softmax	Average pool, 13-d fc, softmax

Pretraining model technology can be used to train a large target network without overfitting when the target dataset is smaller than the base dataset.^47^ In the experiment, we first trained the ResNet based on the ImageNet dataset, and then we transferred it to our target network to be trained on a target dataset. ImageNet is a large-scale hierarchical image database, which contains 3.2 million cleanly annotated images spread over 5247 categories.^48^ It has been the most influential dataset in computer vision.^25,49–51^

## 3 Results and discussion

### 3.1 Spectral analysis of the samples

**Fig. 5:**
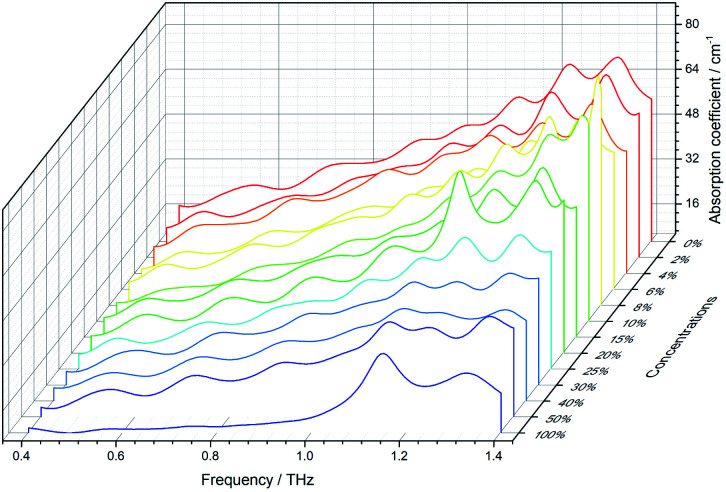
The absorption coefficients of 13 concentration gradients of the samples.

In this paper, the THz absorption coefficients of the samples were obtained using the THz-TDS system. Fig. 5 shows THz absorption coefficients of 13 concentration gradients of the samples in the range of 0.4 THz to 1.4 THz. In Fig. 5, rice is the 0% concentration sample, and carbendazim is the 100% concentration sample. It can be observed that carbendazim exhibits two distinct absorption peaks at 1.15 THz and 1.32 THz. However, as the sample concentration decreases, the absorption peaks of carbendazim become progressively less obvious. Thus, it is hard to accurately distinguish between rice and a low concentration sample by the absorption coefficient. For further study, the samples’ absorption coefficients were translated to two-dimensional images using eqn (3).

### 3.2 The new images generated by the WGAN

**Fig. 6:**
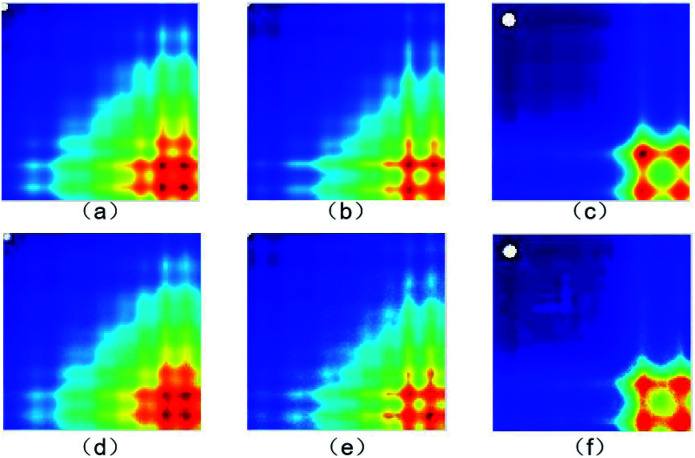
(a)–(c) The actual images and (d)–(f) generated images (0%, 2% and 100% concentration, respectively).

The actual images and generated images are shown in Fig. 6. The actual images (Fig. 6(a)–(c)) are placed in the first row, arranged in the order of 0%, 2% and 100% concentration. The generated images (Fig. 6(d)–(f)) are placed in the second row, arranged in the order of 0%, 2% and 100% concentration. It is clear that the generated images are similar to the actual images.

The structural similarity index (SSIM)^52^ was employed to quantificationally measure the similarity of the generated and actual images. The more similar the generated and actual images, the closer the SSIM value is to 1. For the 0%, 2% and 100% concentration samples, the SSIM values are 0.92, 0.94 and 0.98, respectively. The results mean that the generated images keep the key features of the actual images well.

### 3.3 The identification results of the WGAN-ResNet

**Fig. 7:**
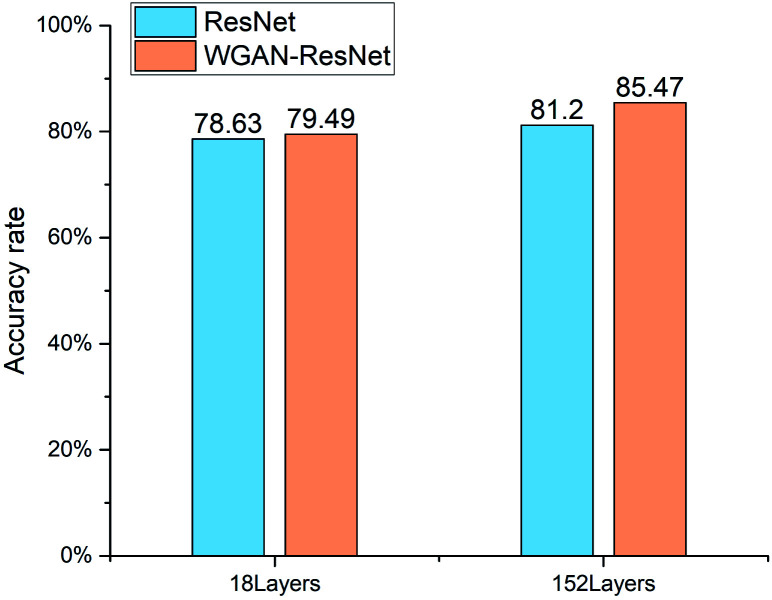
The identification results of the ResNet and WGAN-ResNet.

The identification results of the ResNet and WGAN-ResNet are displayed in Fig. 7. The accuracy rate is defined as follows:10
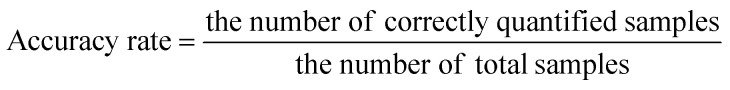


For the ResNet, the quantification accuracy rate of the 152 layer ResNet is 2.57% higher than that of the 18 layer. For the WGAN-ResNet, the quantification accuracy rate of the 152 layer model is 5.98% higher than that of the 18 layer. For both the ResNet and WGAN-ResNet, the 152 layer models have better performances than those of the 18 layer, which is consistent with the previous report.^25^ This indicates that the deeper network has better feature extraction ability.

The 18 layer ResNet has 1.8 GFLOPs, and the 152 layer ResNet has 11.3 GFLOPs.^25^ This means that the model complexity of the 152 layer ResNet is higher than that of the 18 layer ResNet. Thus, training the ResNet models requires a large number of samples. When the ResNet was trained without the WGAN, the number of training samples was only 429. The number of training samples could not meet the needs of model training. In order to make more samples for training the ResNet models, WGAN was employed. The results show that the quantification accuracy rates of WGAN-ResNet are better than ResNet. For the 18 layer model, the quantification accuracy rate of the WGAN-ResNet is 0.86% higher than that of the ResNet. And for the 152 layer model, the identify accuracy rate of the WGAN-ResNet is 4.27% higher than that of the ResNet. This is because the ResNet model parameters were trained sufficiently using the new images generated by the WGAN. This also indicates that the WGAN is a feasible way to augment data for a shortage of learning samples.

To avoid model overfitting, we introduced pretraining model technology to reduce the ResNet model parameters. The task which trains the ResNet based on the ImageNet dataset is called task A. Task B is the task that trained the ResNet based on our dataset. The network AnB_ResNet_18 is a network architecture based on the 18 layer ResNet, and the model parameters of the first *n* layers are copied from task A and frozen, while the parameters of the remaining 5 – *n* layers are randomly initialized and trained based on task B. The network AnB_ResNet_152 is a network architecture based on the 152 layer ResNet, and the model parameters of the first *n* layers are copied from task A and frozen, while the parameters of the remaining 5 – *n* layers are randomly initialized and trained based on task B. To train the ResNet networks, we used the Adam method with a learning rate of 1*e*^−4^ and a batch size of 128.

**Fig. 8:**
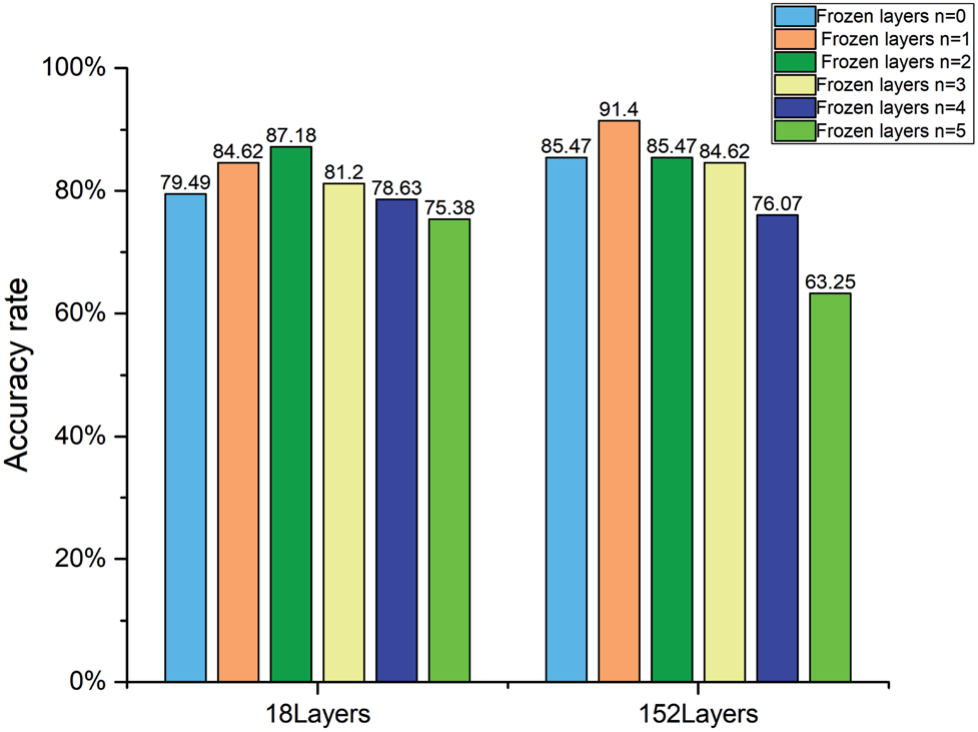
Accuracy rate of the 18 layer and 152 layer WGAN-ResNet with different numbers of frozen layers.

The quantification accuracy rates with different numbers of frozen layers are displayed in Fig. 8. When the frozen layer *n* is 0, it represents that the model parameters in the five layers shown in Table 1 will change with training. When the frozen layer *n* is 1, the first layer conv1 shown in Table 1 is frozen. And when the frozen layer *n* is 2, the first two layers conv1 and conv2_x are frozen, and so on. When the frozen layer *n* is 5, it means that the model parameters in conv1, conv2_x, conv3_x, conv4_x and conv5_x cannot be changed by training. As shown in Fig. 8, the accuracy rate rises as the frozen layers increase at the beginning. However, as the frozen layers increase further, the accuracy rate falls. The features of samples in our dataset are quit different from those in the ImageNet dataset. When the frozen layers increase gradually, the co-adaptation and the feature extraction capability deteriorates. The best accuracy rate is 91.4%, which is obtained by using the 152 layer ResNet with the first layer frozen.

**Fig. 9:**
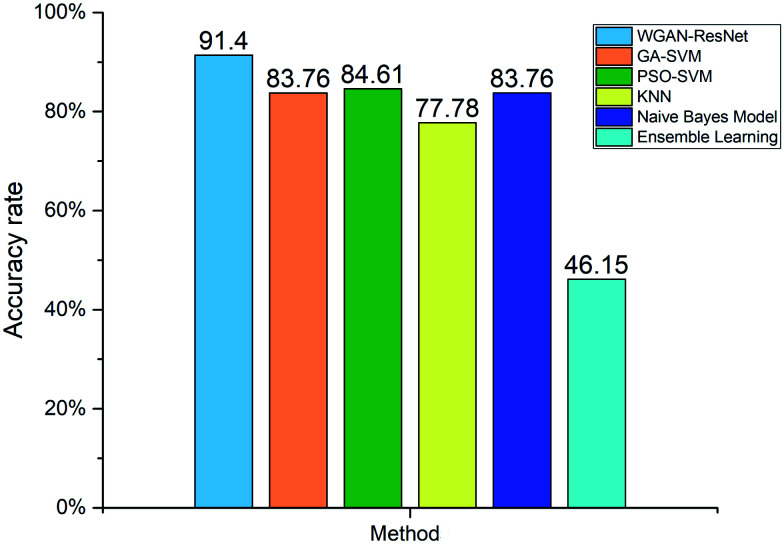
Comparison of the WGAN-ResNet with SVM, KNN, a naïve Bayes model and ensemble learning.

For further analysis, we compared our proposed WGAN-ResNet with a support vector machine (SVM),^53^*k*-nearest neighbor (KNN),^54^ naïve Bayes model^55^ and ensemble learning.^56^ The hyper-parameters of SVM were optimized by a genetic algorithm (GA)^57^ and particle swarm optimization (PSO).^58,59^ SVM, KNN, the naïve Bayes model and ensemble learning can be considered to be shallow learning. The sample features used by shallow learning are low-level features (generally edge texture features). Different from SVM, KNN, the naïve Bayes model and ensemble learning, the ResNet is not only able to extract low-level features, but is also able to extract high-level features.^25^ The high-level features are based on low-level features, which have richer semantic information. The accuracy rates of the above methods are shown in Fig. 9. Our proposed WGAN-ResNet achieved a 91.4% accuracy rate, which is higher than those of the compared methods.

## 4 Conclusion

In this paper, we proposed a new pesticide residue terahertz spectroscopy detection method, which combines two deep learning networks, the WGAN and ResNet, together. Our method demonstrates the potential application of deep learning in the detection of pesticide residues and expands the application of THz spectroscopy. Different from the previous spectral analysis methods, we translated the one-dimensional absorption coefficients into two-dimensional images. To solve the problem of insufficient learning samples, we employed a WGAN to generate new learning samples and pretraining model technology to reduce the training parameters in the ResNet. By using our proposed WGAN-ResNet, the best accuracy rate is 91.4%, which is better than those of SVM, KNN, the naïve Bayes model and ensemble learning. In summary, our proposed method taps into the potential application of deep learning in pesticide residue detection, expanding the application of THz spectroscopy.

Trace detection and the extraction of more features from complex samples will be our future research directions. To extract more features from complex samples, we will use the information fusion method. This fuses more spectrum parameters (such as the refractive index and dielectric constant) together as the input of the WGAN-ResNet. For trace detection, we will enhance the interaction between the pesticide and the terahertz spectrum using metamaterials.

## Author contributions

B. Q. and R. Y. did the terahertz experiments. B. Q and R. Y. provided the funding. B. Q. and Y. L. were the major contributors to writing the manuscript. B. Q. and R. Y. developed the idea and supervised the project. D. Z., Y. G. and J. Z. reviewed and edited the manuscript.

## Conflicts of interest

There are no conflicts to declare.

## Supplementary Material
